# Coping with Workplace Violence against General Practitioners and Nurses in Heilongjiang Province, China: Social Supports and Prevention Strategies

**DOI:** 10.1371/journal.pone.0157897

**Published:** 2016-06-21

**Authors:** Siqi Zhao, Lijun Qu, He Liu, Lijun Gao, Mingli Jiao, Jinghua Liu, Libo Liang, Yanming Zhao, Qunhong Wu

**Affiliations:** 1 Department of Health Policy and Hospital Management, School of Public Health, Harbin Medical University, Harbin, Heilongjiang province, China; 2 Department of ophthalmology, The 2nd Affiliated Hospital of Harbin Medical University, Harbin, Heilongjiang province, China; 3 Department of Social Medicine, School of Public Health, Harbin Medical University, Harbin, Heilongjiang province, China; 4 Institute of Quantitative and Technical Economics, Chinese Academy of Social Science, Beijing, China; 5 Department of general medicine, School of Public Health, Qiqihar Medical University, Qiqihar, Heilongjiang province, China; 6 Department of CT, The 2nd Affiliated Hospital of Harbin Medical University, Harbin, Heilongjiang province, China; Medical University of Vienna, AUSTRIA

## Abstract

The study’s objectives were to: 1) use social support theory to examine factors influencing healthcare workers’ opinions about workplace violence (WPV) prevention strategies, and 2) to determine the types of support that general practitioners (GPs) and general nurses sought and expected to use after WPV exposure. A cross-sectional survey was used to assess a sample of 448 GPs and 412 general nurses from 90 township hospitals located in Heilongjiang province, China. Results revealed that workers exposed to physical, psychological or both WPV types had a strong opinion about the necessity of improving diagnosis/treatment competence, developing violence prevention guidelines and plans, using protective equipment, and reinforcing staff by providing back-up support. The last two strategies were also selected by tertiary hospital workers in our previous study. In addition, workers with high anxiety selected the following prevention strategies as most effective: improving doctor-patient communication skills; installing cameras on wards; keeping work areas bright; improvements in violence reporting, statistics, and interventions; security patrols in the key departments; reinforcing staff; and correcting inaccurate media perspectives and reports. The last four strategies were also selected by tertiary hospital workers. All respondents expected to receive organisational and social support. In conclusion, these prevention strategies should be tailored to the different requirements of specific populations. Furthermore, it is necessary for organisations, the public, and policymakers to provide powerful support in WPV prevention.

## Introduction

Workplace violence (WPV) is a serious global public health problem and has attracted public attention[1, 2]. The World Health Organization (WHO) categorises WPV into two types: (1) physical violence (e.g. beating, kicking, slapping, stabbing, shooting, pushing, biting, and pinching) and (2) psychological violence (e.g. threat of physical force against another person/group that can result in harm to physical, mental, spiritual, moral, or social development)[3]. WPV is common in healthcare settings in China, placing healthcare workers at high risk. These assaults have included events leading to death or serious injury[4]. Since patients and their family members are the main source of violent incidents, this study focuses on these individuals [5].

Particularly in China, most existing research has focused on city rather than township hospitals[6]. However, research in other countries has determined there is higher risk to rural GPs compared to city hospitals[7–9].

In China, township hospitals are primary healthcare institutions that provide basic rural health services for people living in towns and villages. Furthermore, they act as hubs for two-way referral services, potentially decreasing medical resources and associated costs[10].

New healthcare reform in China focuses on activities that strengthen town-level services[11]. A 2013 report by China’s Ministry of Health reported that, of all 37,097 township hospitals, 996 were located in Heilongjiang province and employed 8158 GPs and 3,616 registered nurses [12]. Additionally, because of work characteristics, township GPs and nurses have increased job-related responsibilities[13].

Previous studies have reported that the adverse WPV-related consequences have effects at individual, organisational, and societal levels. At the individual level, healthcare workers who have experienced WPV show signs of depression, anxiety, higher suicide rates, low job satisfaction[5], low work performance efficiency[14], and poor physical health status [15, 16] that influence quality of care provided. These adverse outcomes may also have direct and indirect organisational costs, such as workers leaving the healthcare profession[17] and lower morale[18]. In a WHO research study, it was reported that the result can be reduction in health services for the general population and increased healthcare costs. Particularly in developing countries, equal access to primary healthcare is threatened if already scarce healthcare workers abandon their professions because of the threat of violence[3]. The prevalence of WPV may also affect social stability (e.g. public trust decline and negative social environment).

Studies have found that social support can buffer the effects of violence on health- and work-related outcomes (e.g. high anxiety, job stress and dissatisfaction)[19, 20], thereby diminishing adverse consequences among healthcare workers exposed to WPV. Although researchers define social support multiple ways, the common characteristic is its availability through social relationships connected to external resources. Sarason[21] and Kuichen Yang[22] divided social support into three subsystems to represent the concept: individual, organisational, and social. Social support theory frequently focuses on vulnerable groups (e.g. the elderly, battered women, patients)[23–25]. Since healthcare workers can be regarded as a vulnerable group[26], we based our research on this theoretical framework.

Studies report that enhancing individual competency in ways such as patient communication skill improvement and diagnostic/treatment competence can partly prevent WPV[26, 27]. At an organisational level, enhancing security in high-risk departments (e.g. installing cameras, increasing security patrols) has relieved healthcare workers’ pressure and decreased WPV[28]. In China, some hospitals provide batons and helmets to workers to decrease WPV-related fear; however, this method may not be a permanent solution because it can increase anxiety and carrying inconvenient equipment can affect service quality. At a societal level, many researchers in China promote legislation enactment that protects healthcare workers and the development of authoritative WPV guidelines[29, 30].

Coping with WPV has become an urgent problem requiring investigation. However, in China, most studies focus on risk factors with application to anti-violence strategies without consideration of healthcare workers’ backgrounds or requirements [1, 2].

In our previous study, we researched the factors influencing healthcare workers’ opinions of preventative workplace violence strategies in tertiary hospitals [4]. In the current study, we focus on township hospital GPs and nurses, examine influential factors, and compare population-specific requirements. Another objective is to examine the types of support GPs and general nurses sought and expected to use following WPV exposure.

## Materials and Methods

### Study Design and Population

A retrospective cross-sectional design was used. The survey was conducted in three locations (Harbin, Daqing, and Hegang) in Heilongjiang province, China. In 2012, Heilongjiang had a population of 38.1 million and 996 township hospitals. Resulting from time and resource limitations, 90 township hospitals (approximately 10%) were purposively selected according to geographical location, population, and township health levels. The sample included 30 hospitals in the Harbin area (middle), 30 in Daqing (west), and 30 in Hegang (east), respectively. Permission was obtained from all hospitals.

### Data Collection

The survey was conducted from September 2014–November 2014. Access was negotiated with supervisors from each study hospital. After providing consent, respondents completed and returned an anonymous questionnaire (names/identifiers not requested) to a box in the manager’s office. A total of 990 questionnaires were distributed, and 860 valid questionnaires were returned (total response rate: 90.0%, total valid rate: 96.5%).

### Questionnaire

The questionnaire was developed in 2003 by the International Labor Office (ILO), International Council of Nurses (ICN), the World Health Organization (WHO), and Public Services International (PSI) joint program[3]. First, formal documented permission for questionnaire use was obtained from the ILO and WHO. Subsequently, experts revised questions about violence prevention strategies to fit study objectives and the China township hospital context. The questionnaire was then translated into Chinese and back-translated into English to verify its accuracy. The 18 experts included epidemiologists, health service management experts, public health specialists, and experts from administrative departments. A two-week test-retest reliability check (*r* = 0.87) was conducted with 30 participants who were then excluded from the study. Data were collected using the final questionnaire, which consisted of the following four sections:

1. Demographic and workplace data: age, gender, years of experience, profession, and professional title. One question measuring WPV anxiety was measured on a scale ranging from 1 (*not at all*) to 5 (*extremely high*).
2. Experience of physical violence within the past 12 months (defined as intentionally physically violent behaviour). Events were measured by asking, ‘Have you experienced physical violence in the past 12 months?’ Participants responded ‘Yes’ or ‘No’.
3. Experience of psychological violence within the past 12 months, including verbal abuse (humiliating or degrading behaviour, lack of respect), threatening events (offensive behaviour or comments that represent threats or elicit fear), and sexual harassment (unwelcome behaviour or comments of a sexual nature). Psychological abuse was measured with the question, ‘Have you experienced verbal abuse, threatening events, and/or sexual harassment in the past year?” Participants responded ‘Yes’ or ‘No’.
4. Perception of effective strategies for WPV prevention. Fourteen items (strategies) were provided as options (e.g. improving doctor-patient communication skills, security patrols, enacting WPV legislation).

### Data Analyses

Descriptive statistics were calculated to summarise the healthcare workers’ demographic characteristics and frequency of support strategy use. Pearson’s chi-squared test was used to analyse differences in violence exposure based on characteristics. Multiple logistic regression was used to determine what participant characteristics predicted WPV prevention strategy choice. The data were analyzed using IBM SPSS Statistics 19.0, and p < 0.05 was considered statistically significant.

### Ethical Approval

The Research Ethics Committee of Harbin Medical University provided ethical approval before data collection commenced, and all procedures were approved by each study hospital. All participants provided informed consent in writing when submitting questionnaires.

## Results

### Respondents’ Demographic Characteristics

A total of 860 out of 990 questionnaires were returned from 448 GPs and 412 general nurses. Most respondents were male (n = 500; 58.1%) and 57.9% of participants were between the ages of 36 to 45 years (n = 498), while 25.6% participants were over 45 years of age (n = 220). A total of 30 (3.5%) participants reported experiencing exposure only to physical violence within the past 12 months, 257 (29.9%) reported only psychological violence exposure, and 76(8.8%) reported exposure to both types of violence.

Characteristics of the respondents with violence exposure are presented in Table 1. The majority of workers who exposed to physical violence were female (n = 16; 4.4%), had fewer than 10 years of experience (n = 8; 6.8%), or had a junior professional title(n = 9; 4.1%). The majority of participants who exposed to psychological violence were general nurses (n = 135; 32.8%) or worked in shifts (n = 125; 32.2%). The majority of participants who were exposed to both types of violence were general nurse (n = 42; 10.2%) or had a junior professional title (n = 25; 11.3%).

**Table 1:** Frequency distributions for characteristics of healthcare workers exposed to physical, psychological and both types of violence.

Characteristics	Physical violence only				Psychological violence only				Both types of violence			
	*N*^a^	%	*χ*^*2*^	*p*	*N*^b^	%	*χ*^*2*^	*p*	*N*^*c*^	%	*χ*^*2*^	*p*
Gender												
Males	14	2.8	1.62	0.26	126	25.2	12.51	0.001	76	15.2	-	-
Female	16	4.4			131	36.4			0	0		
Age												
≤35	16	11.3	31.59	0.00	61	43	15.9	0.00	12	8.5	8.81	0.01
36–45	12	2.4			144	28.9			34	6.8		
>45	2	0.9			52	23.6			30	13.6		
Years of experience												
≤10	8	6.8	4.4	0.11	47	39.8	8.21	0.02	4	3.4	6.73	0.04
11–20	12	3			122	30.3			34	8.5		
>20	10	2.9			88	25.9			38	11.2		
Profession												
General practitioner	6	1.3	12.83	0.00	122	27.2	3.14	0.09	34	7.6	1.81	0.19
General nurse	24	5.8			135	32.8			42	10.2		
Professional title												
Senior	4	1.8	2.37	0.31	75	34.4	7.63	0.02	24	11.0	5.92	0.05
Intermediate	17	4			107	25.5			27	6.4		
Junior	9	4.1			75	33.8			25	11.3		
Anxiety level												
Extremely high	0	0	20.75	0.00	24	27	22.29	0.00	12	13.5	28.4	0.00
High	8	7.5			28	26.2			22	20.6		
Moderate	12	6.8			32	18.2			16	9.1		
Low	2	0.5			136	37.4			18	4.9		
Zero	8	6.5			37	29.8			8	6.5		
Shift work												
Yes	2	0.5	18.56	0.00	125	32.2	1.84	0.18	44	11.3	5.5	0.02
No	28	5.9			132	28			32	6.8		

Note: *N*^a^ = 30(workersexposed to physical violence); *N*^b^ = 257 (workers exposed to psychological violence); *N*^c^ = 76(workers exposed to both types of violence)

Physical violence exposure significantly differed by age, profession, anxiety level and shift work status. Psychological violence exposure differed significantly by gender, age, years of experience, professional title, and anxiety level. Exposure to both types of violence differed significantly by age, years of experiences, anxiety level and shift work status.

### Healthcare Workers’ Use of and Expectations about Support Strategies

Table 2 shows the types of support that GPs and general nurses sought after violence exposure. Types of support received were classified as individual or organisational subsystems. Following only physical violence exposure, the highest proportion of GPs (90.0%) reported relaying on their own strength, and general nurses primarily relied on family support (83.8%) (individual supports).The highest proportion of GPs(90.0%) received organisational support by completing an accident/injury report, and most general nurses most relied on reporting the incident to a leader(90.0%).

**Table 2:** Methods of obtaining support after exposure to violence.

Support	Method of Obtaining Support after Exposure to WPV	*N*^a^(%)		*N*^b^(%)		*N*^c^(%)	
		general practitioners	*general nurses*	general practitioners	*general nurses*	general practitioners	*general nurses*
Individual	Relay on their own strength	27(90.0)	24(80.0)	174(67.7)	221(86.0)	70(92.1)	66(86.8)
	Talk with co-workers	17(56.7)	20(66.7)	213(82.9)	232(90.3)	64(84.2)	58(76.3)
	Support from family	26(86.7)	25(83.3)	210(81.7)	198(77.0)	69(90.8)	67(88.2)
	Support from psychologist	8(26.7)	9(30.0)	185(72.0)	177(68.9)	66(86.8)	62(81.6)
Organisational	Financial compensation	20(66.7)	18(60.0)	59(23.0)	34(13.2)	65(85.5)	66(86.8)
	Complete accident/injury report	27(90.0)	26(86.7)	18(7.0)	52(20.2)	61(80.3)	57(75.0)
	Change department	15(50.0)	10(33.3)	8(3.1)	162(63.0)	33(43.4)	35(46.1)
	Report to leader	16(53.3)	27(90.0)	167(65.0)	186(72.4)	61(80.3)	63(82.9)

Note: *N*^a^ = 30(workers exposed to physical violence); *N*^b^ = 257 (workers exposed to psychological violence); *N*^c^ = 76(workers exposed to both types of violence)

After psychological violence exposure, the highest proportion of GPs and general nurses attained individual support by talking with co-workers(82.9% and 90.3%, respectively) For organisational support, the highest proportion reported the incident to their leader(65.0% and 72.4%. respectively).

After exposure to both types of violence, the highest proportion of GPs (92.1%) reported relaying on their own strength, and general nurses primarily relied on family support (88.2%) (individual supports).The highest proportion of GPs and general nurses received organisational support by receiving financial compensation (85.5% and 86.8, respectively).

When evaluating overall organisational support, most respondents who experienced only physical, only psychological and both types of WPV exposure reported feeling very dissatisfied (55.7%,57.4% and 60.7%, respectively).

Table 3 shows the supports that GPs and general nurses indicated they expected to use if exposed to WPV. Options were divided into three support subsystems (individual, organisational, and social). The social support subsystem included obtaining help through government, legislation, and labour organisations (e.g. Medical Association or Staff Safety Association). Respondents expected organisational and social support subsystems regardless of violence type experienced and their profession.

**Table 3:** Healthcare workers’ expected use of support subsystems.

Support Subsystem	*N*^a^(%)		*N*^b^(%)		*N*^c^(%)	
	general practitioners	*general nurses*	General practitioners	*general nurses*	general practitioners	*general nurses*
Individual support subsystem	11(36.7)	14(46.7)	144(56.0)	125(48.6)	46(60.5)	41(53.9)
Organisational support subsystem	26(86.7)	28(93.3)	217(84.4)	229(89.1)	71(93.4)	69(90.8)
Social support subsystem	24(80.0)	22(73.3)	223(86.8)	232(90.3)	68(89.5)	68(89.5)

Note: *N*^a^ = 30(workers exposed to physical violence); *N*^b^ = 257 (workers exposed to psychological violence); *N*^c^ = 76(workers exposed to both types of violence).

### Multiple Logistic Regression of GPs and General Nurses’ Opinions of Violence Prevention Strategies

To examine factors influencing the choices of the most useful strategies for WPV prevention, respondents’ characteristics and WPV exposure experience were used in multiple logistic regression analysis. Anti-violence strategies were divided into individual, organisation, and social levels based on support source.

As shown in Table 4, at the individual level, using protective equipment (e.g. batons, helmets) and improved diagnosis/treatment competence had three common factors: workers only exposed to physical violence, psychological violence, and both WPV types. These factors also influenced the following strategies at the organisational level: staff reinforcement, camera installation, using bright lights at night, promoting fee transparency, and fee payment assurance. At the social level, developing violence prevention guidelines and plans were considered useful in WPV prevention.

**Table 4:** Factors influencing healthcare workers’ choices of strategies for workplace violence prevention.

Dimensions	Strategies	Variables	*OR*	95% CI	*P*
Individual	Use batons, helmets, and other protective equipment	**Age**	0.389	0.252–0.601	0
		**Years of experience**	4.324	2.856–6.547	0
		**Professional title**	1.628	1.371–1.932	0
		**Working rotating shifts**	0.316	0.216–0.462	0
		**Violence type**			
		physical violence only	3.904	1.596–9.549	0.003
		psychological violence only	2.593	1.739–3.868	0
		both types of violence	6.279	3.502–11.257	0
	Improve competence in diagnosis and treatment	**Gender**			
		Male	1.401	1.032–1.902	0.03
		**Violence**			
		physical violence only	5.764	1.917–17.333	0.002
		psychological violence only	1.683	1.217–2.327	0.002
		both types of violence	1.89	1.096–3.261	0.022
	Improve doctor-patient communication skills	**Anxiety level**	1.539	1.294–1.831	0
		**Violence**			
		physical violence only	-	-	-
		psychological violence only	2.575	1.629–4.0071	0
		both types of violence	-	-	-
Organisational	Target training to strengthen competence in responding to violence	**Gender**			
		Male	1.609	1.181–2.191	0.003
		**Years of experience**	1.516	1.062–2.163	0.022
		**Professional title**	0.826	0.710–0.960	0.013
		**Violence**			
		physical violence only	-	-	-
		psychological violence only	1.94	1.391–2.707	0
		both types of violence	-	-	-
	Hospital improvements in violence reporting, statistics, and interventions	**Gender**			
		Male	1.645	1.196–2.262	0.002
		**Years of experience**	1.675	1.186–2.366	0.003
		**Anxiety level**	1.174	1.038–1.328	0.011
		**Working rotating shifts**	0.714	0.53–0.963	0.027
		**Professional title**	1.343	1.160–1.555	0
	Security patrols in the emergency or other key departments	**Violence**			
		physical violence only	2.381	1.059–5.354	0.036
		psychological violence only	-	-	-
		both types of violence	1.174	1.038–1.233	0.017
		**Gender**			
		Male	2.213	1.608–3–046	0
		**Years of experience**	1.73	1.195–2.503	0.004
		**Professional title**	0.819	0.705–0.951	0.009
		**Profession**			
		General practitioner	0.596	0.432–0.822	0.002
		**Anxiety level**	1.151	1.003–1.319	0.044
		**Violence**			
		physical violence only	-	-	-
		psychological violence only	-	-	-
		both types of violence	5.864	2.058–16.709	0.001
	Reinforce staff with back-up support	**Age**	0.381	0.253–0.574	0
		**Years of experience**	3.423	2.344–4.998	0
		**Professional title**	1.477	1.262–1.729	0
		**Profession**			
		General practitioner	0.629	0.453–0.873	0.006
		**Working rotating shifts**	0.533	0.381–0.744	0
		**Anxiety level**	1.308	1.143–1.497	0
		**Violence**			
		physical violence only	4.465	1.849–10.782	0.001
		psychological violence only	2.207	1.534–3.174	0
		both types of violence	2.027	1.151–3.568	0.014
	Improve the treatment process and shorten wait times	**Age**	0.399	0.265–0.601	0
		**Gender**			
		Male	1.611	1.177–2.205	0.003
		**Years of experience**	2.833	1.948–4.122	0
		**Professional title**	1.211	1.047–1.400	
		physical violence only	-	-	-
		psychological violence only	1.799	1.281–2.525	0.001
		both types of violence	-	-	-
	Install cameras on wards, keep work areas bright by using lights at night	**Years of experience**	1.553	1.100–2.192	0.012
		**Profession**			
		General practitioner	0.714	0.531–0.960	0.026
		**Anxiety level**	1.307	1.152–1.483	0
		**Violence**			
		physical violence only	2.512	1.040–6.066	0.041
		psychological violence only	1.797	1.296–2.491	0
		both types of violence	3.356	1.821–6.188	0
	Promote transparency of fees, assure fee payment	**Age**	0.553	0.365–0.839	0.005
		**Professional title**	1.222	1.048–1.425	0.011
		**Working rotating shifts**	0.58	0.422–0.797	0.001
		**Violence**			
		physical violence only	2.572	1.061–6.233	0.036
		psychological violence only	5.544	3.805–8.078	0
		both types of violence	13.77	6.263–30.264	0
Social	Police officers stationed in the hospital	**Age**	0.379	0.253–0.567	0
		**Gender**			
		Male	1.795	1.266–2.545	0.001
		**Years of experience**	3.544	2.434–5.160	0
		**Professional title**	1.225	1.047–1.435	0.012
		**Working rotating shifts**	0.415	0.300–0.575	0
		**Violence**			
		physical violence only	3.366	1.403–8.076	0.007
		psychological violence only	-	-	-
		both types of violence	1.977	1.155–3.384	0.013
	Enact workplace violence legislation	**Age**	0.422	0.281–0.634	0
		**Years of experience**	2.473	1.714–3.569	0
		**Working rotating shifts**	0.456	0.335–0.622	0
		**Violence**			
		physical violence only	-	-	-
		psychological violence only	2.14	1.533–2.988	0
		both types of violence	24.68	8.666–70.325	0
	Develop violence prevention guidelines and plans	**Age**	0.421	0.283–0.626	0
		**Gender**			
		Male	1.874	1.365–2.573	0
		**Years of experience**	2.259	1.582–3.226	0
		**Violence**			
		physical violence only	3.244	1.329–7.918	0.01
		psychological violence only	1.912	1.376–2.655	0
		both types of violence	6.967	3.564–13.622	0
	Accurate perspectives and reports by media, promote respect of medical workers	**Age**	0.558	0.377–0.826	0.004
		**Years of experience**	1.551	1.086–2.216	0.016
		**Working rotating shifts**	0.543	0.102–0.733	0
		**Anxiety level**	1.299	1.142–1.478	0
		**Violence**			
		physical violence only	-	-	-
		psychological violence only	2.868	2.028–4.057	0
		both types of violence	-	-	-

Compared to general nurses, GPs thought that reinforcing staff with back-up support was the least useful strategy. Healthcare workers’ with high anxiety levels consider the following strategies effective: improving doctor-patient communication skills; improving violence reporting, statistics, and interventions; security patrols, camera installation, using bright lights at night; staff reinforcement, and accurate media reporting.

Workers with many years of experience and high professional titles indicated the following strategies would be useful: using protective equipment, improving violence reporting, statistics, and interventions; staff reinforcement; improving treatment and shortening wait times; and police officers stationed in hospital.

## Discussion

### The Importance of Organisational and Social Support

Our findings indicated that GPs and general nurses received little organisational support following WPV exposure and support sources were generally individual (personal or from those with close relationships).The healthcare workers who have only exposed to psychological violence sought for help from co-workers. Previous studies have confirmed that talk with co-workers can release their tension gain from WPV[20]. However, all workers exposed to WPV expected more organisational and social supports. A supportive workplace atmosphere influences feelings of being cared for and increases confidence when experiencing WPV and related adverse consequences [31, 32]. Therefore, organisational support for WPV-exposed workers should be increased by actively reporting violence, supporting compensation efforts, and expressing intention for departmental change.

The public should also be aware that healthcare workers are a vulnerable group, and corresponding policy and legislation should be implemented to strengthen protection and increase perpetrator penalties. Consequently, health organisations and the media can positively influence a supportive environment for WPV-exposed workers [33–35].

### Workers’ Choices for Useful Violence Prevention Strategies

Healthcare workers in our study revealed useful anti-violence prevention and support strategies from individual, organisational, and social levels. Our multivariate logistic regression analysis indicated that different groups have different prevention strategy requirements.

At the individual level, healthcare workers with any type of WPV exposure considered improving diagnosis/treatment competence and using protective equipment to be effective. In our previous study, healthcare workers in tertiary hospitals with both exposure types only chose the second strategy as individually useful [4]. This suggests that healthcare workers in township hospitals focus on individual factors compared to workers in tertiary hospitals.

Studies have shown that both education level and GP and general nurse diagnosis/treatment competence are lower in townships than urban hospitals[36, 37]. Consistently, it was reported that this lack of competence was associated with anger among patients and family members[38]. Likewise, our results verified that WPV-exposed township hospital healthcare workers attended more to increasing their diagnosis and treatment abilities. However, most township hospitals in China have not established a training and continuing education system, and many researchers indicate that more opportunities should be provided to township GPs and general nurses[39, 40].

Since WPV is unexpected and instantaneous, it is easy to understand why healthcare workers desired protective equipment to protect physical safety, regardless of previous type of WPV exposure. In China, some hospitals already provide this equipment to workers to alleviate WPV-related fear.

At the organisational level, staff reinforcement, camera installation, bright night work areas, promoting fee transparency, and fee payment assurance are potentially useful strategies. Similarly, in our previous tertiary hospital study, workers exposed to both violence types also indicated that reinforcing staff was useful [4].

Township hospitals are basic medical and health institutions, and have heavy workloads and a high level of responsibilities. Studies have confirmed that increased workload and staff shortages were the most common reasons for WPV [41], and Chinese township are particularly prone to staff shortages [42] Our findings indicate that compared with general nurses, GPs identified staff reinforcement as significantly less useful. However, the proportion of GPs is higher than general nurses in township hospitals (more than 2:1). In contrast, in Canada and Japan, the number of GPs is lower than nurses (1:4 and 1:5, respectively) [43]

Since nurses have increased long-term patient contact and consistently high workload, there is a need for increased collaboration in the provision of healthcare services[44]. Other than the above factors, years of experience, professional title, and anxiety level also influence need for this strategy. Specificallyworkers with increased experience and higher anxiety more frequently emphasised collaboration.

The problem of non-transparent and non-standardised charges is problematic in Chinese township hospitals [45] particularly because services are provided to residents in poor financial situations. Raising medical expenses or confusing prices may both increase patients’ economic burden and create doctor-patient conflict, facilitating WPV. Consistently, research indicates patients may commit violence as the results of lack of transparency and feeling of being cheated[46]. This verifies our findings that fee transparency promotion and fee payment assurance are useful prevention strategies.

Installing cameras on wards and using lights at night can also be viewed as viable security measures. Studies have noted that working in a dark environment at night was a high-risk factor predicting WPV-exposure[28]. Furthermore, the Occupational Safety and Health Administration (OSHA) requires camera installation and constant use in high-risk areas [47]. Two influential background factors affecting workers’ opinions about this prevention strategy were years of experience and anxiety level.

WPV-exposed workers had strong opinions about developing violence prevention guidelines and plans. Specifically, guidelines can increase knowledge about coping with WPV, and violence awareness as well as effective protection skills. Likewise, prevention plans can purposively organize anti-violence activities establish organisational prevention mechanisms. Although no professional Chinese institution has established systematic guidelines or WPV prevention programs, the OSHA published prevention guidelines that have become a gold standard for guiding healthcare worker practices. In 2014, a large medical bulletin-board system, Dingxiangyuan, published a Chinese version of violence prevention guidelines for healthcare workers to provide authoritative professional guidelines. These combined OSHA guidelines and the reality of the current healthcare system. However, the Chinese guidelines almost always emphasise self defence, without requiring organisational strategies or equipment provision (e.g. training opportunities or alarm installation).

Our findings indicate that anxiety levels might have affected healthcare workers’ strategy choices. At the organisational level, improvements in violence reporting, statistics, and interventions; security patrols; and reinforcing staff were strongly associated with anxiety. At the societal level, accurate media perspectives and reports were important. In our previous tertiary hospital study, workers with high anxiety also chose these strategies[4]. However, unlike the previous study, at the individual level, healthcare workers in township hospitals with high anxiety selected patient communication skill improvement.

Overall, the strategies selected by healthcare workers represent the three support subsystems. Thus, township hospital workers seem to believe that WPV prevention requires a combination of individual, organisational, and societal efforts. Considering these expectations and the prevalence of WPV, organisations and society should increase the focus on WPV prevention.

## Limitations

As the result of time and resource limitations, only 90 hospitals participated, limiting the generalisability of findings to healthcare workers in township hospitals in the Heilongjiang province. Additionally, data was retrospectively collected and could suffer from recall bias. However, our prevention strategy research was based on social support theory and may be valuable for controlling WPV and drawing public and policymaker attention to the necessity of adequately supporting healthcare workers.

## Conclusions

This study’s results could assist with developing appropriate policies and strategies to contend with the perpetuation of WPV against healthcare workers in township hospitals. Strategies should be based on differing requirements of specific populations. Healthcare workers with any type of WPV-exposure indicated that improved diagnosis/treatment competence, use of protective equipment, staff reinforcement, promotion of fee transparency, camera installation, staff reinforcement, bright work areas, and violence prevention guidelines were useful prevention strategies. To reduce anxiety, many of the above strategies in addition to improved doctor-patient communication skills; improved violence reporting, statistics, and interventions; security patrols; and correction of inaccurate media perceptions and reporting may be successful. In a future study, we will examine the utility of these chosen strategies in decreasing WPV.

## Abbreviations

GPs: General practitioners
ICN: International Council of Nurses
ILO: International Labor Office
OSHA: Occupational Safety and Health Administration
PSI: Public Services International
WPV: Workplace violence
WHO: World Health Organization
